# Clinical and pathological significance of ROS1 expression in intrahepatic cholangiocarcinoma

**DOI:** 10.1186/s12885-015-1737-4

**Published:** 2015-10-16

**Authors:** Kyung-Hun Lee, Kyoung-Bun Lee, Tae-Yong Kim, Sae-Won Han, Do-Youn Oh, Seock-Ah Im, Tae-You Kim, Nam-Joon Yi, Kwang-Woong Lee, Kyung-Suk Suh, Ja-June Jang, Yung-Jue Bang

**Affiliations:** 1 Department of Internal Medicine, Seoul National University Hospital, Seoul, Republic of Korea; 2 Cancer Research Institute, Seoul National University College of Medicine, Seoul, Republic of Korea; 3 Department of Pathology, Seoul National University Hospital, 101 Daehak-ro, Jongno-gu Seoul, 110-744 South Korea; 4 Department of Surgery, Seoul National University Hospital, Seoul, Republic of Korea

**Keywords:** ROS1, Biliary tract cancer, Cholangiocarcinoma, Immunohistochemistry, FISH

## Abstract

**Background:**

More knowledge about genetic and molecular features of cholangiocarcinoma is needed to develop effective therapeutic strategies. We investigated the clinical and pathological significance of ROS1 expression in intrahepatic cholangiocarcinoma.

**Methods:**

One hundred ninety-four patients with curatively resected intrahepatic cholangiocarcinoma were included in this study. Tumor tissue specimens were collected and analyzed for ROS1 gene rearrangement using fluorescence in situ hybridization (FISH) and ROS1 protein expression using immunohistochemistry (IHC).

**Results:**

ROS1 immunohistochemistry was positive (moderate or strong staining) in 72 tumors (37.1 %). ROS1 protein expression was significantly correlated with well differentiated tumors, papillary or mucinous histology, oncocytic/hepatoid or intestinal type tumors, and periductal infiltrating or intraductal growing tumors (vs. mass-forming cholangiocarcinoma). ROS-expressing tumors were associated with better disease-free survival (30.1 months for ROS1 expression (+) tumors vs. 9.0 months for ROS1 (−) tumors, *p* = 0.006). Moreover, ROS1 expression was an independent predictor of better disease-free survival in a multivariate analysis (HR 0.607, 95 % CI 0.377–0.976; *p* = 0.039). Although break-apart FISH was successfully performed in 102 samples, a split pattern indicative of ROS1 gene rearrangement was not found in the examined samples.

**Conclusion:**

ROS1 protein expression was associated with well-differentiated histology and better survival in our patients with resected intrahepatic cholangiocarcinoma. ROS1 gene rearrangement by break-apart FISH was not found in the examined samples.

## Background

Biliary tract cancer (BTC) is an aggressive disease with a very poor prognosis with a median survival of less than 1 year [1]. It is a heterogeneous group of disease including intrahepatic, perihilar, or distal cholangiocarcinoma and gallbladder cancer, with diverse epidemiology, etiology, and pathogenesis. Among them, intrahepatic cholangiocarcinoma is a distinct disease with increasing incidence in the western countries and worldwide, and its etiology and molecular pathogenesis differs from the other BTCs [2]. Five-year survival rate after curative surgery for intrahepatic cholangiocarcinoma remains poor ranging 20–32 %, and this is poorer than that for hilar cholangiocarcinoma (30–42 %), and for distal cholangiocarcinoma (18–54 %) [3–5]. Understanding its molecular features and developing new effective strategies are urgent and important; however, the molecular and genetic features of BTCs have been inadequately investigated in comparison to other common solid malignancies.

ROS1 is a receptor tyrosine kinase (RTK) oncogene that activates the SH2 domain tyrosine phosphatases SHP-1 and SHP-2, the mitogen-activated protein kinase ERK1/2, insulin receptor substrate 1 (IRS-1), phosphatidylinositol 3-kinase (PI3K), protein kinase B (AKT), STAT3 and VAV3 signaling pathways [6]. The expression of ROS1 was found in human cancers of the central nervous system, stomach, liver, kidney, and colon [6]. Moreover, gene rearrangement of ROS1 has been found in nonsmall cell lung cancer (NSCLC) [7–11], glioblastoma multiforme [12], gastric cancer [13], and colon cancer [14]. These rearrangements create fusion proteins in which the kinase domain of ROS1 becomes constitutively active and drives cellular proliferation. Crizotinib, an oral MET/anaplastic lymphoma kinase (ALK) inhibitor, has shown encouraging clinical activity in ROS1-rearranged NSCLC, indicating that ROS1 rearrangement is a driver mutation in NSCLC [8, 15, 16]. Thus, the activity of crizotinib is of significant interest for the treatment of ROS1-rearranged tumors.

Recently, Gu et al. found a fusion of the ROS1 gene with the FIG gene in 2 out of 23 patients (8.7 %) with cholangiocarcinoma; the authors suggested that this could be a driver mutation, because it confers transforming activity to bile duct cells and can be effectively blocked with an ROS1 tyrosine kinase inhibitor [17]. Indeed, cholangiocarcinoma with ROS1 gene fusion would be a good candidate for treatments targeting ROS1 such as crizotinib; however, the actual incidence and clinical significance of ROS1 rearrangements in BTC have not been fully known.

We aimed to investigate both protein expression and gene fusion of ROS1 in a larger number of patients with intrahepatic cholangiocarcinoma. Immunohistochemistry and fluorescence in situ hybridization (FISH) analysis were performed and correlated with clinicopathologic features.

## Methods

### Patients and clinicopathologic parameters

Patients who underwent curative surgery for intrahepatic cholangiocarcinoma at Seoul National University Hospital, Seoul, Republic of Korea, from 1992 to 2010, and had available medical records and formalin-fixed paraffin blocks of tumor were eligible for analysis. Clinical information including age, sex, size of tumor, and surgical methods was collected from the medical records; pathologic information including differentiation, histologic type, gross type, vascular invasion, and perineural invasion was collected form pathology reports and slide review. Criteria for pT (pathologic T stage) followed the intrahepatic bile duct tumor staging of American Joint Committee on Cancer 7^th^ edition [18] Tumor differentiation was categorized based on the grading system described by the World Health Organization classification [19]. Adjuvant chemotherapy and/or radiotherapy were at the physician’s discretion considering histology and lymph node involvement. This study was carried out in compliance with the Helsinki Declaration and approved by the Institutional Review Board of Seoul National University Hospital (H-1011-046-339). Informed consent was waived by the Institutional Review Board of Seoul National University Hospital.

### Construction of tissue microarray and immunohistochemical staining

Suitable areas with two representative tumor areas for each case were marked on the H&E stained sections, then core tissue specimens (2 mm in diameter) were collected from individual paraffin-embedded tissues and rearranged in new tissue array blocks by using a trephine apparatus (SuperBioChips Laboratories, Seoul, Korea). Each tissue microarray had four cores of normal liver, normal bile duct, and normal gastrointestinal tract mucosa as internal controls. Sections (4 μm) were stained for ROS1 (ROS1(D4D6) rabbit monoclonal antibody, cat. number #3287 1:10 dilution, Cell Signaling Technology, Beverly, MA) after an antigen retrieval process using Bond Epitope Retrieval Solution 2 at 99 °C for two minutes (Leica Biosystems, Wetzlar, Germany). The slides were automatically stained using Bond-Max IHC and ISH slide stainer and a Bond Polymer Refine Detection Kit (Leica Biosystems, Wetzlar, Germany).

### Evaluation of immunohistochemistry

Positive staining for ROS1 was observed in cytoplasm; the intensity of staining was graded as negative; no staining in any cellular component, weak (1+); faint staining in cytoplasm, moderate (2+); unequivocal positive staining with negative background staining, or strong (3+); strong cytoplasmic staining with negative background staining. Because stained pattern was not patched but usually diffuse, we could not separately evaluate the area of positive cells, but 5 % of tumor cells was used as a cutoff value for positive staining. For comparative analysis of protein expression and clinicopathologic parameters, dichotomized values such as positive and negative were used and the criteria of positivity was ≥2+ intensity in ≥5 % of tumor cells.

### ROS1 break-apart fluorescence in situ hybridization (FISH) assay

Break-apart FISH probe consisted of the distal part of Exon 30 of ROS1(6q22) (RH104060-SHGC-14420)) directly labeled with PlantinumBrightTM550 (red signal) and the proximal part of Exon 42 of ROS1(6q22) (RH69070-RH68126) directly labeled with PlatinumBrightTM495 (green signal) (Repeat-FreeTM PseidonTM ROS1 (6q22) Break probe, KBI-10752, Kreatech Diagnostics, Amsterdam, Netherlands). Briefly, 2 micrometer sections were deparaffinized and dehydrated. The slides were treated with pretreatment reagent (Abott Molecular, Des Plaines, IL) at 80 °C for 40 min and reacted with protease powder (Abott Molecular, Des Plaines, IL) in protease buffer at room temperature after HCL and microwave treatment. The probe set was applied and incubated in ThermoBrite (Abbott Molecular, Des Plaines, IL) at 80 °C for 10 min to denature the probes followed by incubation at 37 °C for 16 h to allow hybridization. The samples were analyzed using an X100 oil immersion lens on an Olympus BX-51TRE microscope (Olympus, Tokyo, Japan) equipped with DAPI, green, orange, aqua, and triple-pass (DAPI/Green/Orange) filters (Abbott-Vysis). At least 50 nuclei per sample were assessed. Signals were evaluated as: (a) no gene rearrangement on either chromosome, i.e. two sets of separate red and green signals, (b) gene rearrangement on one chromosome, i.e. one combined signal and one separate red and green signal, and (c) deletion of the distal portion of ROS1 as indicated by one combined signal and a single green signal found in >15 % of tumor cells [9]. Specimen from non-small cell lung cancer with ROS1 fusion was used as a positive control.

### Statistical analysis

Comparative analysis of clinicopathologic parameters was evaluated using the chi-squared (*χ*^2^) test or Fisher’s exact test. Survival analysis was performed using Kaplan-Meier analysis and Cox’s proportional hazard model. Disease-free survival (DFS) was defined as the time from the surgery until the patient survives without any signs or symptoms of the cancer. Overall survival (OS) was defined as the time to any cause of death. The results were considered statistically significant when p values were < 0.05. All tests were performed using IBM SPSS version 21.

## Results

### Patient demographics

**Fig. 1:**
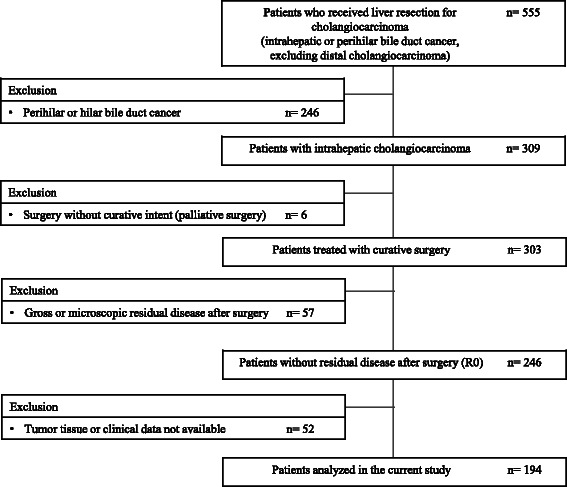
Selection of patients. This diagram summarized the selection of patients included in the study. Out of 555 patients who received liver resection for cholangiocarcinoma, 194 patients with curatively resected intrahepatic cholangiocarcinoma were included in the analysis

The number of patients who received liver resection for intrahepatic cholangiocarcinoma was 309, and excluding those whose surgery was not curative or R0 resection and those whose tumor tissue or clinical data were not available, 194 patients were finally included in the current study (Fig. 1). The demographic characteristics of the patients are summarized in Table 1. Briefly, 149 (76.8 %) of patients were male, and the median age of the entire population was 62 years. With regard to the known underlying liver disease and etiology of cholangiocarcinoma, 15 patients had chronic hepatitis, 12 of whom had hepatitis B virus infection and 3 had hepatitis C virus infection; three patients had infection of clonorchis sinensis; and 3 patients had hepatolithiasis.

**Table 1:** ROS1 expression and clinicopathologic parameters

ROS1 expression		N (%)	Negative	Positive	*p*.
			122 (62.9)	72 (37.1)	
Sex (194)	Male	149 (76.8)	96 (64.4)	53 (35.6)	0.418
	Female	45 (23.2)	26 (57.8)	19 (42.2)	
Age (yr) (194)	62 [37–89]		60.1 ± 9.0	61.5 ± 10.4	0.347
Size (cm) (185)	5.0 [0.3–26.0]		5.5 ± 2.9	5.6 ± 3.9	0.673
Operation (183)	Lobectomy	129 (70.5)	84 (65.1)	45 (34.9)	0.635
	Segmentectomy	54 (29.5)	32 (59.3)	22 (40.7)	
Number (188)	Single	169 (89.9)	107 (63.3)	62 (36.7)	0.114
	Multiple	19 (10.1)	14 (73.7)	5 (26.3)	
Gross type (189)	Mass forming	133 (70.4)	91 (68.4)	42 (31.6)	0.006*
	Periductal infiltrating	10 (5.3)	5 (50.0)	5 (50.0)	
	Intraductal polypoid	24 (12.7)	8 (33.3)	16 (66.7)	
	Mixed	22 (11.6)	16 (72.7)	6 (27.3)	
Extent of tumor (188)	Confined liver	92 (48.9)	50 (54.3)	42 (45.7)	0.005*
	Extrahepatic invasion	96 (51.1)	71 (74.0)	25 (26.0)	
pT stage (190)	Tis	1 (0.5)	1 (100)	0 (0.0)	0.226
	T1	86 (45.3)	52 (60.5)	34 (39.5)	
	T2a	39 (20.5)	21 (53.8)	18 (46.2)	
	T2b	12 (6.3)	9 (75.0)	3 (25.0)	
	T3	50 (26.3)	37 (74.0)	13 (26.0)	
	T4	2 (1.1)	1 (50.0)	1 (50.0)	
pN stage (106)	pN0	61 (57.5)	35 (57.4)	26 (42.6)	0.462
	pN1	45 (42.5)	29 (64.4)	16 (35.6)	
Resection margin (177)	R0	151 (85.3)	100 (66.2)	51 (33.8)	0.400
	R1-2	26 (14.7)	15 (57.7)	11 (42.3)	
Lymphatic invasion (187)	Absent	117 (62.6)	71 (60.7)	46 (39.3)	0.278
	Present	70 (37.4)	48 (68.6)	22 (31.4)	
Vascular invasion (180)	Absent	127 (70.6)	81 (63.8)	46 (36.2)	0.595
	Present	53 (29.4)	36 (67.9)	17 (32.1)	
Neural invasion (184)	Absent	137 (74.5)	89 (65.0)	48 (35.0)	0.687
	Present	47 (25.5)	29 (61.7)	18 (38.3)	
Differentiation (194)	Well	34 (17.5)	14 (41.2)	20 (58.8)	0.150
	Moderate	112 (57.7)	75 (67.0)	37 (33.0)	
	Poor	48 (24.7)	33 (68.8)	15 (31.3)	
Histologic subtype (194)	Adenocarcinoma, tubular	167 (86.1)	111 (66.5)	56 (33.5)	0.009*
	Papillary or mucinous carcinoma	15 (7.7)	4 (26.7)	11 (73.3)	
	Others^a^	12 (6.2)	7 (58.3)	5 (41.7)	
Cell type (172)	Nonintestinal type	137 (79.7)	92 (67.2)	45 (32.8)	0.019*
	Intestinal type	35 (20.3)	19 (54.3)	19 (54.3)	
Progression (194)	No evidence of disease	62 (32.0)	34 (54.8)	28 (45.2)	0.024*
	Recurrence or metastasis	119 (61.3)	83 (69.7)	36 (30.3)	
	Censored	13 (6.7)	5 (38.5)	8 (61.5)	
Death (194)	Alive	60 (30.9)	32 (53.3)	28 (46.7)	0.176
	Deceased	126 (64.9)	85 (67.5)	41 (32.5)	
	Censored	8 (4.1)	5 (62.5)	3 (37.5)	

Median [range]; mean ± sd

*p < 0.05

^a^Undifferentiated carcinoma, adenosquamous carcinoma, mixed adenocarcinoma and neuroendocrine carcinoma

Most patients received lobectomy or hemihepatectomy of the liver (*n* = 129, 66.5 %), followed by segmentectomy (*n* = 54, 28.1 %) and others (*n* = 11, 5.7 %). Tumor size ranged from 0.3 to 26.0 cm (mean tumor size 5 cm), and 19 patients (10.1 %) had more than 1 tumor in the liver. Lymph node involvement by the tumors was found in 45 patients among 106 patients whose lymph nodes were resected and assessed. Histological subtypes of resected tumors included adenocarcinoma in 167 (86.1 %), papillary or mucinous carcinoma in 15 (7.7 %), and others in 12 (6.2 %). Tumors were classified as well-differentiated in 34 (17.5 %), moderately-differentiated in 112 (57.7 %), and poorly-differentiated in 48 (24.7 %). Adjuvant treatment was given to 25 patients (12.9 %), of whom 13 chemotherapy, 1 radiotherapy, and 11 concurrent chemoradiation.

After median follow-up period of 30.0 months (range 1–196) after surgery, 119 (61.3 %) patients had recurrent disease, and 62 patients remained disease-free. One hundred twenty-six (64.9 %) patients had deceased at the time of analysis.

### Immunohistochemical analysis and break-apart fluorescence in situ hybridization of ROS1

**Fig. 2:**
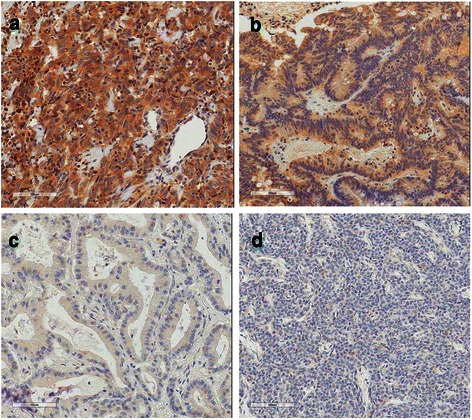
​Immunohistochemical staining for ROS1. Tumor sections were stained for ROS1 with rabbit monoclonal antibody (D4D6), purchased from Cell Signaling Technology, Beverly, MA. The intensity of cytoplasmic staining was graded as (a) strong (3+); strong cytoplasmic staining with negative background staining, (b) moderate (2+); unequivocal positive staining with negative background staining, (c) weak (1+); faint staining in cytoplasm, or (d) negative; no staining in any cellular component. The photographs were taken at a magnification of x200

Representative expression patterns of ROS1 are presented in Fig. 2. Positive ROS1 staining was observed in the cytoplasm in a diffuse pattern. In 70 samples (36.1 %) staining was moderate, while in two samples (1.0 %) staining was strongly positive; in 62 samples (32.0 %) showed weak intensity and remaining 60 cases (30.9 %) were negatively stained (Table 2). FISH was performed in 194 samples, and its signal was detected in 102 samples, but evidence of gene rearrangement (split pattern using break-apart FISH) was not found in the examined samples.

**Table 2:** The positivity of ROS1 by immunohistochemistry

ROS1 staining	Number	Percent		Number	Percent
0	60	30.9 %	Negative	122	62.9 %
1+	62	32.0 %			
2+	70	36.1 %	Positive	72	37.1 %
3+	2	1.0 %			
Total	194	100 %	Total	194	100 %

### Correlation of ROS1 expression and clinicopathological features of the patients

Clinicopathologic features listed according to ROS1 expression level are summarized in Table 1. The group with positive staining included 72 moderate to strongly stained samples (37.1 %).

ROS1-expressing tumors were more frequent in periductal (50.0 %) or intraductal type (66.7 %) than mass forming type (31.6 %) as characterized according to the histologic subtypes proposed by the Liver Cancer Study Group of Japan [20, 21]. Microscopic features of tumors with ROS1 expression were significantly related to well differentiated histology, papillary or mucinous tumors, and intestinal type. In addition, the stage at the time of surgery was lower in ROS1-expressing tumors, with a higher proportion of T1 or T2 stage tumors as compared to later stage tumors and less invasion into adjacent organs. The expression of ROS1 did not significantly correlate with known risk factors or etiologies of cholangiocarcinoma, or the adjuvant treatments.

### Univariate and multivariate survival analysis of ROS1 expression

**Fig. 3:**
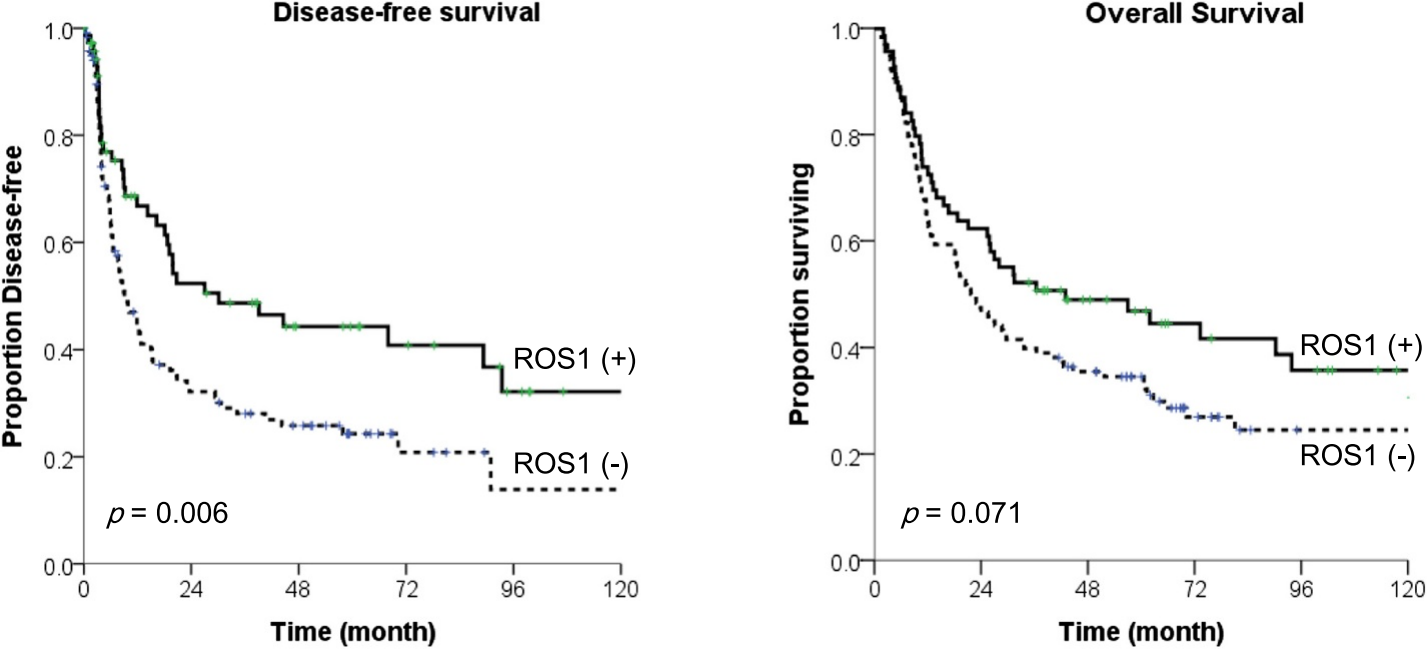
Kaplan-Meier curves for disease-free survival and overall survival according to ROS1 expression. ROS-expressing tumors were associated with better disease-free survival (30.1 months for ROS1 expression (+) tumors vs. 9.0 months for ROS1 (−) tumors, *p* = 0.006). Also, patients with ROS1 (+) tumors had better overall survival, not reaching statistical significance (*p* = 0.071)

ROS1-expressing tumors were associated with better disease-free survival (Fig. 3). Median disease-free survival for ROS1-expressing (+) tumors and non-expressing (−) tumors was 30.1 months and 9.0 months, respectively (*p* = 0.006). Median overall survival was 43.0 months and 21.7 months, respectively (*p* = 0.071).

As extensive lymph node dissection is not routinely performed during curative surgery of intrahepatic cholangiocarcinoma, we confined the multivariate survival analysis to the patients without lymph node metastasis. With covariates including tumor size, gross appearance, multiplicity of tumors, tumor histology, differentiation, and the presence of vascular, neural, or lymphatic invasion, ROS1 expression was an independent predictor of longer disease-free survival (HR 0.607, 95 % CI 0.377–0.976; *p* = 0.039, Table 3).

**Table 3:** Disease free survival and ROS1 expression

		Univariate		Multivariate	
		Median(m)	*p.*	HR [95 % CI]	*p.*
ROS1 expression	Negative *vs.* positive	9 *vs.* 30	0.006	0.58 [0.37–0.89]	0.014
Size of tumor	Cm	1.053^a^	0.008	1.06 [1.01–1.11]	0.019
Number of tumor	Single *vs.* multiple	15 *vs*. 4	0.001	2.27 [1.24–4.16]	0.008
Gross type	Mass forming *vs.* periductal	9 *vs*. 89	<0.001	0.48 [0.24–0.98]	0.042
Differentiation	Well *vs.* moderate to poor	89 *vs.* 9	<0.001	2.22 [1.09–4.51]	0.028
Perineural invasion	Absent *vs.* present	19 *vs.* 9	0.039	1.63 [1.04–2.55]	0.034
Vascular invasion	Absent vs present	15 *vs*. 8	0.051		
Lymphatic invasion	Absent vs present	19 *vs*. 8	0.009		
Histologic type	Papillary/mucinous vs.	NA *vs*. 11	0.026		
	Tubular/undifferentiated				

^a^Mean hazard ratio by Cox proportional hazard model; HR, hazard ratio; CI, confidential interval

## Discussion

Molecular pathogenesis of intrahepatic cholangiocarcinoma is of particular importance, not only because it is fatal disease with increasing incidence, but also little has been known compared to other common cancers [2]. EGF, HGF/MET, VEGF, KRAS/MAPK, and IL-6/STAT pathways have been found to be deregulated in cholangiocarcinoma, but no effective therapies targeting these pathways have been developed. There is an eager need for more knowledge and clinical application in the field of this disease. As rearrangement of ROS1 gene was reported in cholangiocarcinoma recently [17, 22], and ROS1 inhibitors such as crizotinib or foretinib (GSK1363089) have shown remarkable activity in ROS1-driven tumors [8, 16, 23], the actual incidence of protein expression and gene rearrangements of ROS1, as well as its clinical significance in BTC, were pursued in the present study.

ROS1 was discovered more than 30 years ago as an oncogene, but it is one of the last few remaining orphan receptor tyrosine kinases with an as yet unidentified ligand, and its normal functions have not been fully identified so far [24]. It is expressed in human cancers such as glioblastoma, and cancers of stomach, liver, kidney and colon [6]. Wild-type ROS1 has been shown to have transformative activity with downstream signaling of the SH2 domain tyrosine phosphatases SHP-1 and SHP-2, the mitogen-activated protein kinase ERK1/2, insulin receptor substrate 1 (IRS-1), phosphatidylinositol 3-kinase (PI3K), protein kinase B (AKT), STAT3 and VAV3 signaling pathways [6]. Importantly, fusion proteins created by the rearrangements also have the kinase domain of ROS1 and it becomes constitutively active and drives cellular proliferation. Both wild-type and rearranged ROS1 have transformative activity attributable to its kinase domain.

Fusion of ROS1 gene was first found in 2 out of 23 BTCs (8.7 %) [17], but there have been scarce reports following the original study. Recently, ROS1 alterations were found in 1 out of 100 (1 %) patients with intrahepatic cholangiocarcioma [25]. In another report, ROS1 fusion was found in 14–16 % of patients with gallbladder carcinoma or extrahepatic cholangiocarcinoma, but not those with intrahepatic cholangiocarcinoma [26]. We could not find any ROS1 rearrangement by FISH in 102 Korean patients with intrahepatic cholangiocarcinoma. The actual incidence of ROS1 rearrangement in intrahepatic cholangiocarcinoma is expected to be lower than that previously reported due to ethnic and environmental differences [27].

Although ROS1 gene rearrangements were not found, ROS1 protein expression was found in significant portion of BTCs. Interestingly, it was related to specific characteristics of tumors and better disease-free survival. ROS1 expression was more common in well-differentiated tumors than in moderately- or poorly-differentiated tumors. Specifically, papillary or mucinous carcinomas were strongly related to expression of the ROS1 protein (73.3 %). The gross appearance of cholangiocarcinoma is divided into three types: mass-forming, periductal infiltrating, intraductal growth type [20, 21]. The mass-forming subtype is the most common and spreads via venous and lymphatic vessels, exhibiting poorer prognosis [28, 29]. ROS1-expressing tumors were periductal or intraductal (50 % and 33.3 %, respectively) rather than mass forming (31.6 %). In general, ROS1 expression is related to less aggressive tumors, well differentiated features, and better survival in BTC.

ROS1 expression was also a predictor of favorable survival in NSCLC, as well as BTC. In a large cohort of 1478 NSCLCs, ROS1 expression was correlated with better survival and specific features such as low T stages, TTF1 and napsin expression, and certain histomorphological adenocarcinoma patterns (lepidic, acinar, and solid) [30], although there is also a contradictory data [31]. Moreover, gastric adenocarcinomas expressing ROS1 by immunohistochemistry tended to present with differentiated tumors and lower lymph node status [13]. Similar results were found in breast cancer with regard to histologic grade, mitotic count, estrogen receptor expression, and Ki-67 proliferation index [32]. The pathogenic role of ROS1 is suggested in these specific tumor types as in BTCs.

ROS1 expression as determined by IHC was moderate to strong in 38 % of tumors, but gene rearrangement assessed by FISH was not found in our patients. This is in contrast to the previous reports in NSCLC, where IHC and FISH results were strongly correlated. The sensitivity and specificity of ROS1 IHC for rearrangements by FISH is reported to be more than 90 % [33–35] and, as such, IHC is suggested as an effective screening tool in NSCLC. The threshold level for ROS1 positive expression in IHC differs among reports, but 2+ or moderate expression is usually considered to be positive. In contrast, a significant portion of cholangiocarcinoma samples exhibited greater than moderate expression of ROS1, yet we could not find any ROS1 gene rearrangement by FISH. Therefore, we conclude that ROS1 IHC cannot be used as a screening tool for ROS1 rearrangement in BTC.

As protein expression of ROS1 does not directly indicate fusion and activation of the ROS1 gene in BTCs, we should be cautious in selecting treatment strategies for these tumors. As the expression of ROS1 was related not only to gene rearrangements, but also to other biological processes as epigenetic changes [31], further research on biological and clinical role of ROS1 expression is warranted. Inhibition of both ROS1 and its frequent fusion partner FIG in the HuCCT1 cell line, which expresses ROS1 protein, led to decreased cell proliferation, although the existence of FIG-ROS1 fusion protein was not specified in the article [36]. More data regarding biological and clinical role of ROS1 expression, as well as the effects of specific inhibitors of ROS1 in BTCs are needed.

## Conclusion

ROS1 protein expression was associated with well-differentiated histology and better survival in patients with resected intrahepatic cholangiocarcinoma. ROS1 gene rearrangement by break-apart FISH was not found in the examined samples.

## References

[1] Khan SA, Taylor-Robinson SD, Toledano MB, Beck A, Elliott P, Thomas HC (2002). Changing international trends in mortality rates for liver, biliary and pancreatic tumours. J Hepatol.

[2] Patel T (2014). New insights into the molecular pathogenesis of intrahepatic cholangiocarcinoma. J Gastroenterol.

[3] Nakagohri T, Kinoshita T, Konishi M, Takahashi S, Gotohda N (2008). Surgical outcome and prognostic factors in intrahepatic cholangiocarcinoma. World J Surg.

[4] Hirano S, Kondo S, Tanaka E, Shichinohe T, Tsuchikawa T, Kato K, Matsumoto J, Kawasaki R (2010). Outcome of surgical treatment of hilar cholangiocarcinoma: a special reference to postoperative morbidity and mortality. J Hepatobiliary Pancreat Sci.

[5] Sakamoto Y, Kosuge T, Shimada K, Sano T, Ojima H, Yamamoto J, Yamasaki S, Takayama T, Makuuchi M (2005). Prognostic factors of surgical resection in middle and distal bile duct cancer: an analysis of 55 patients concerning the significance of ductal and radial margins. Surgery.

[6] Acquaviva J, Wong R, Charest A (2009). The multifaceted roles of the receptor tyrosine kinase ROS in development and cancer. Biochim Biophys Acta.

[7] Rikova K, Guo A, Zeng Q, Possemato A, Yu J, Haack H, Nardone J, Lee K, Reeves C, Li Y (2007). Global survey of phosphotyrosine signaling identifies oncogenic kinases in lung cancer. Cell.

[8] Bergethon K, Shaw AT, Ou SH, Katayama R, Lovly CM, McDonald NT, Massion PP, Siwak-Tapp C, Gonzalez A, Fang R (2012). ROS1 rearrangements define a unique molecular class of lung cancers. J Clin Oncol.

[9] Takeuchi K, Soda M, Togashi Y, Suzuki R, Sakata S, Hatano S, Asaka R, Hamanaka W, Ninomiya H, Uehara H (2012). RET, ROS1 and ALK fusions in lung cancer. Nat Med.

[10] Rimkunas VM, Crosby KE, Li D, Hu Y, Kelly ME, Gu TL, Mack JS, Silver MR, Zhou X, Haack H (2012). Analysis of receptor tyrosine kinase ROS1-positive tumors in non-small cell lung cancer: identification of a FIG-ROS1 fusion. Clin Cancer Res.

[11] Govindan R, Ding L, Griffith M, Subramanian J, Dees ND, Kanchi KL, Maher CA, Fulton R, Fulton L, Wallis J (2012). Genomic landscape of non-small cell lung cancer in smokers and never-smokers. Cell.

[12] Charest A, Lane K, McMahon K, Park J, Preisinger E, Conroy H, Housman D (2003). Fusion of FIG to the receptor tyrosine kinase ROS in a glioblastoma with an interstitial del(6)(q21q21). Genes Chromosomes Cancer.

[13] Lee J, Lee SE, Kang SY, Do IG, Lee S, Ha SY, Cho J, Kang WK, Jang J, Ou SH (2013). Identification of ROS1 rearrangement in gastric adenocarcinoma. Cancer.

[14] Aisner DL, Nguyen TT, Paskulin DD, Le AT, Haney J, Schulte N, Chionh F, Hardingham J, Mariadason J, Tebbutt N (2014). ROS1 and ALK fusions in colorectal cancer, with evidence of intratumoral heterogeneity for molecular drivers. Mol Cancer Res.

[15] Shaw AT, Ou SH, Bang YJ, Camidge DR, Solomon BJ, Salgia R, Riely GJ, Varella-Garcia M, Shapiro GI, Costa DB (2014). Crizotinib in ROS1-rearranged non-small-cell lung cancer. N Engl J Med.

[16] Davies KD, Doebele RC (2013). Molecular pathways: ROS1 fusion proteins in cancer. Clin Cancer Res.

[17] Gu TL, Deng X, Huang F, Tucker M, Crosby K, Rimkunas V, Wang Y, Deng G, Zhu L, Tan Z (2011). Survey of tyrosine kinase signaling reveals ROS kinase fusions in human cholangiocarcinoma. PLoS One.

[18] Edge SB, Compton CC (2010). The American Joint Committee on Cancer: the 7th edition of the AJCC cancer staging manual and the future of TNM. Ann Surg Oncol.

[19] Nakanuma Y, Sato Y, Harada K, Sasaki M, Xu J, Ikeda H (2010). Pathological classification of intrahepatic cholangiocarcinoma based on a new concept. World J Hepatol.

[20] Yamasaki S (2003). Intrahepatic cholangiocarcinoma: macroscopic type and stage classification. J Hepatobiliary Pancreat Surg.

[21] Guglielmi A, Ruzzenente A, Campagnaro T, Pachera S, Valdegamberi A, Nicoli P, Cappellani A, Malfermoni G, Iacono C (2009). Intrahepatic cholangiocarcinoma: prognostic factors after surgical resection. World J Surg.

[22] Saborowski A, Saborowski M, Davare MA, Druker BJ, Klimstra DS, Lowe SW (2013). Mouse model of intrahepatic cholangiocarcinoma validates FIG-ROS as a potent fusion oncogene and therapeutic target. Proc Natl Acad Sci U S A.

[23] Davare MA, Saborowski A, Eide CA, Tognon C, Smith RL, Elferich J, Agarwal A, Tyner JW, Shinde UP, Lowe SW (2013). Foretinib is a potent inhibitor of oncogenic ROS1 fusion proteins. Proc Natl Acad Sci U S A.

[24] El-Deeb IM, Yoo KH, Lee SH (2011). ROS receptor tyrosine kinase: a new potential target for anticancer drugs. Med Res Rev.

[25] Graham RP, Barr Fritcher EG, Pestova E, Schulz J, Sitailo LA, Vasmatzis G, et al. Fibroblast growth factor receptor 2 translocations in intrahepatic cholangiocarcinoma. Hum Pathol. 2014. 10.1016/j.humpath.2014.03.014 24837095

[26] Peraldo Neia C, Cavalloni G, Balsamo A, Venesio T, Napoli F, Sassi F, Martin V, Frattini M, Aglietta M, Leone F (2014). Screening for the FIG-ROS1 fusion in biliary tract carcinomas by nested PCR. Genes Chromosomes Cancer.

[27] Liu P, Wu Y, Sun L, Zuo Q, Shi M (2013). ROS kinase fusions are not common in Chinese patients with cholangiocarcinoma. Nan Fang Yi Ke Da Xue Xue Bao.

[28] Blechacz B, Komuta M, Roskams T, Gores GJ (2011). Clinical diagnosis and staging of cholangiocarcinoma. Nat Rev Gastroenterol Hepatol.

[29] Razumilava N, Gores GJ (2013). Classification, diagnosis, and management of cholangiocarcinoma. Clin Gastroenterol Hepatol.

[30] Warth A, Muley T, Dienemann H, Goeppert B, Stenzinger A, Schnabel PA, et al. ROS1 expression and translocations in non-small-cell lung cancer: clinicopathological analysis of 1478 cases. Histopathology. 2014. 10.1111/his.12379 24456475

[31] Lee HJ, Seol HS, Kim JY, Chun SM, Suh YA, Park YS, Kim SW, Choi CM, Park SI, Kim DK (2013). ROS1 receptor tyrosine kinase, a druggable target, is frequently overexpressed in non-small cell lung carcinomas via genetic and epigenetic mechanisms. Ann Surg Oncol.

[32] Eom M, Lkhagvadorj S, Oh SS, Han A, Park KH (2013). ROS1 expression in invasive ductal carcinoma of the breast related to proliferation activity. Yonsei Med J.

[33] Sholl LM, Sun H, Butaney M, Zhang C, Lee C, Janne PA, Rodig SJ (2013). ROS1 immunohistochemistry for detection of ROS1-rearranged lung adenocarcinomas. Am J Surg Pathol.

[34] Mescam-Mancini L, Lantuejoul S, Moro-Sibilot D, Rouquette I, Souquet PJ, Audigier-Valette C, Sabourin JC, Decroisette C, Sakhri L, Brambilla E (2014). On the relevance of a testing algorithm for the detection of ROS1-rearranged lung adenocarcinomas. Lung Cancer.

[35] Yoshida A, Tsuta K, Wakai S, Arai Y, Asamura H, Shibata T, Furuta K, Kohno T, Kushima R (2014). Immunohistochemical detection of ROS1 is useful for identifying ROS1 rearrangements in lung cancers. Mod Pathol.

[36] Deng G, Hu C, Zhu L, Huang F, Huang W, Xu H, Nie W (2014). Downregulation of ROS-FIG inhibits cell proliferation, colonyformation, cell cycle progression, migration and invasion, while inducing apoptosis in intrahepatic cholangiocarcinoma cells. Int J Mol Med.

